# Facile synthesis of Fe-based metal–organic frameworks from Fe_2_O_3_ nanoparticles and their application for CO_2_/N_2_ separation

**DOI:** 10.3762/bjnano.15.74

**Published:** 2024-07-19

**Authors:** Van Nhieu Le, Hoai Duc Tran, Minh Tien Nguyen, Hai Bang Truong, Toan Minh Pham, Jinsoo Kim

**Affiliations:** 1 Faculty of Chemical Engineering, Industrial University of Ho Chi Minh City, 12 Nguyen Van Bao, Go Vap, Ho Chi Minh City 70000, Vietnam; 2 Optical Materials Research Group, Science and Technology Advanced Institute, Van Lang University, Ho Chi Minh City 700000, Vietnam; 3 Faculty of Applied Technology, School of Technology, Van Lang University, Ho Chi Minh City 700000, Vietnam; 4 Department of Chemical Engineering (Integrated Engineering), Kyung Hee University, 1732 Deogyeong-daero, Giheung-gu, Yongin-si, Gyeonggi-do 17104, Korea

**Keywords:** CO_2_/N_2_ separation, Fe_2_O_3_ nanoparticles, hydrothermal reaction, IAST-predicted CO_2_/N_2_ selectivity, MIL-100(Fe)

## Abstract

A facile approach was employed to fabricate MIL-100(Fe) materials from Fe_2_O_3_ nanoparticles through a conventional hydrothermal reaction without the presence of HF and HNO_3_. Effects of trimesic acid content in the reaction system on the quality and CO_2_/N_2_ separation performance of the as-prepared MIL-100(Fe) samples were investigated. Using 1.80 g of trimesic acid in the reaction system yielded the sample M-100Fe@Fe_2_O_3_#1.80, which proved to be the optimal sample. This choice struck a balance between the amount of required trimesic acid and the quality of the resulting material, resulting in a high yield of 81% and an impressive BET surface area of 1365.4 m^2^·g^−1^. At 25 °C and 1 bar, M-100Fe@Fe_2_O_3_#1.80 showed a CO_2_ adsorption capacity of 1.10 mmol·g^−1^ and an IAST-predicted CO_2_/N_2_ selectivity of 18, outperforming conventional adsorbents in CO_2_/N_2_ separation. Importantly, this route opens a new approach to utilizing Fe_2_O_3_-based waste materials from the iron and steel industry in manufacturing Fe-based MIL-100 materials.

## Introduction

Metal-organic frameworks (MOFs) are well-ordered porous hybrid structures assembled from the fundamental components of metal ion clusters and organic linkers. MOFs are well known as multipurpose materials that serve a broad range of applications because of their unique construction variants, enormous surface areas, high thermal stability, changeable pore system sizes, and customizable chemical surfaces [1–6]. The family tree of MOFs holds giant lineages such as Zeolitic Imidazolate Framework (ZIF), University of Olso (UiO), Material of Institute Lavoisier (MIL), Dresden University of Technology (DUT), and others. Among them, the Fe-based MIL-100(Fe) material stands out as an exceptional member of the MIL family because of its distinct properties [7–8]. MIL-100(Fe) offers a substantial number of unsaturated metal sites. These sites act as Lewis acid sites once ligands (–OH and water) are removed from the termini of iron octahedra in the secondary building units (SBUs) through an activated-thermal process in a vacuum-controlled environment [9–10]. Notably, density and oxidation states of the formed unsaturated Fe sites (Fe(II) and Fe(III)) are temperature-dependent, influencing the strength of the Lewis acid properties. Lower temperatures (approx. 150 °C) favor Fe(III), while higher temperatures up to 250 °C promote Fe(II) [9–10]. The MIL-100(Fe) structure is built from oxo-trimers of iron octahedra as SBUs together with bridges of benzene-1,3,5-tricarboxylate, yielding a hybrid supertetrahedral structure. Following that, the hybrid structures self-assemble in a certain sequence to form a topology resembling the MTN-type of zeolite seen in Zeolite Socony Mobil Thirty-Nine (ZSM-39) [11]. The MIL-100(Fe) structure has a cubic series containing two types of large chambers with average diameters of 2.5 and 2.9 nm, which may accommodate guest molecules entering through pentagon (0.47–0.55 nm) and hexagon (0.86 nm) apertures; this is an extremely porous channel system with immense surface area and pore volume [10,12]. Furthermore, MIL-100(Fe) is also widely recognized for its low manufacturing cost, environmental friendliness, and biodegradability. Because of the abovementioned advantages, MIL-100(Fe) has gained popularity as a candidate for gas separation of CO/CO_2_ [9,13], CO/N_2_ [14–15], CO_2_/CH_4_ [16], CH_4_/C_2_H_6_/C_3_H_8_ [17], and SF_6_/N_2_ [18].

In the early stage, MIL-100(Fe) was produced using metallic iron and iron salts, along with HF and HNO_3_, in a conventional hydrothermal reactor operated at high temperatures [9–10]. While HF and HNO_3_ improve yield and quality of MIL-100(Fe), they are harmful to the environment because of their toxicity and corrosiveness. As a result, scientists are consistently working to eliminate HF, HNO_3_, and other enhancing agents from the reaction process while maintaining the material quality. Initially, trimethyl benzene-1,3,5-tricarboxylate and iron(III) chloride hexahydrate (FeCl_3_·6H_2_O) were combined to create MIL-100(Fe) without using HF and HNO_3_. This method resulted in high-quality MIL-100(Fe) with a Langmuir surface area of 2800 m^2^·g^−1^, but the yield was only 47%. In addition, it extended the reaction time to three days and significantly increased the cost because of the more expensive trimethyl benzene-1,3,5-tricarboxylate compared to trimesic acid [19]. These factors caused an immense rise in production cost. Further efforts were made to enhance both product quality and yield by using FeCl_3_·6H_2_O and iron(III) nitrate nonahydrate (Fe(NO_3_)_3_·9H_2_O) as iron precursors in a high-temperature reactor without HF and HNO_3_. The findings revealed that a high Fe(NO_3_)_3_·9H_2_O concentration in the MIL-100(Fe) preparation resulted in excellent outcomes, achieving approximately 80% yield and a BET surface area of 1800 m^2^·g^−1^. These results closely matched those from the original recipe, which utilized metallic iron in the presence of HF and HNO_3_, yielding 82% and a BET surface area of 2050 m^2^·g^−1^ [20]. Since then, various green synthetic routes from ferric nitrate and metallic iron powder have been developed for generating MIL-100(Fe), including low-temperature synthesis [21], solvent-free synthesis [22], and dry gel conversion pathway [23]. It should be noted that the observed BET surface area fluctuates between 1100 and 2100 m^2^·g^−1^, reflecting the quality of the obtained MIL-100(Fe); it depends on the iron precursors, synthetic pathways, and the purification procedure to eliminate unexpected components inside the pore system [20,24–25].

Additionally, iron oxides were considered as iron precursors required to make MIL-100(Fe). The first candidate Fe_3_O_4_ was used to successfully fabricate MIL-100(Fe) in a hydrothermal reactor in the absence of HF and HNO_3_ [26–27]; it resulted in enhanced porosity of the obtained material when increasing the mass ratio between Fe_3_O_4_ and benzene-1,3,5-tricarboxylic acid in the reactor. Very recently, Freund et al. [28] employed Fe_2_O_3_ to produce successfully MIL-100(Fe) in a conventionally hydrothermal reaction at 150 °C for 27 h with the assistance of HF and HNO_3_, which are hazardous chemicals to the environment. In contrast to metallic iron and iron salts, iron oxides are acknowledged for their easy storage and abundant availability in raw materials derived from natural iron oxidation processes and steelmaking industry waste [29–30]. These characteristics play a crucial role in the selection of economical raw materials for the large-scale production of MIL-100(Fe). Utilizing iron oxide as an iron precursor for manufacturing MIL-100(Fe) contributes to the expansion of raw material options. According to what we know, until now, there has been no report on a pathway for generating MIL-100(Fe) from Fe_2_O_3_ without the assistance of HF and HNO_3_.

In this study, MIL-100(Fe) nanoparticles were successfully made utilizing a green synthetic route to convert Fe_2_O_3_ in a traditional hydrothermal reaction with the assistance of trimesic acid. A variety of analytical methods were employed to estimate quality and yield of the as-prepared MIL-100(Fe) materials, including thermogravimetric analysis (TGA), Fourier-transform infrared (FTIR) spectroscopy, powder X-ray diffraction (PXRD) measurements, determination of textural properties, scanning electron microscopy (SEM), and X-ray photoelectron spectroscopy (XPS). Subsequently, the gas separation performance of the as-prepared MIL-100(Fe) samples was assessed by studies regarding CO_2_ and N_2_ adsorption isotherms at various temperatures with pressures up to 1 bar.

## Experimental

### Materials

Iron(III) oxide (Fe_2_O_3_, 96%) and benzene-1,3,5-tricarboxylic acid (H_3_BTC, 95%) were supplied from Sigma-Aldrich. Anhydrous ethanol (EtOH, 99.5%) was acquired from Daejung Chemicals (Korea). Deionized (DI) water was generated using the Aqua Max ultra 360 system from Young-Lin (Korea). No further purification was performed on the chemicals before use.

### Preparation of M-100Fe@Fe_2_O_3_ and MIL-100(Fe) materials

The Fe-based metal-organic framework (MIL-100) was fabricated through a hydrothermal reaction system using iron(III) oxide as the precursor and H_3_BTC as linker for Fe ion clusters. The procedure was adapted from the method described by Aslam and coworkers [26]. Typically, 0.9 g Fe_2_O_3_ and different amounts of H_3_BTC, alongside 45 mL of DI water, were introduced into a Teflon beaker and gently stirred for 15 min at room temperature. The mixture was then carefully sealed inside an autoclave made of stainless steel before being placed inside an electrically heated oven operated at 160 °C for 12 h. Once the resultant slurry had cooled to ambient temperature, it was washed with DI water and EtOH at 60 °C three times. Finally, the solid powder was collected after centrifugation and drying at 70 °C for 12 h. The resulting products were marked as M-100Fe@Fe_2_O_3_#0.90, M-100Fe@Fe_2_O_3_#1.35, M-100Fe@Fe_2_O_3_#1.80, and M-100Fe@Fe_2_O_3_#2.25, corresponding to H_3_BTC amounts of 0.90, 1.35, 1.80, and 2.25 g, respectively.

A MIL-100(Fe) reference sample was prepared in accordance with the protocol described in Horcajada's report [11], with additional details provided in Supporting Information File 1.

### Characterizations

The materials’ crystalline structure was identified via room-temperature powder X-ray diffraction (PXRD) patterns from a MiniFlex600 system (Rigaku, Japan). The scan covered a 2θ range of 3–40° at a speed of 6°·min^−1^. Information regarding the morphologies of Fe_2_O_3_ and M-100Fe@Fe_2_O_3_ materials was collected using a field-emission scanning electron microscope Leo-Supra 55 (Carl Zeiss STM, Germany). An attenuated total reflectance (ATR) setup was adopted to record the Fourier-transform infrared (FTIR) spectra of all samples on a Frontier spectrometer (PerkinElmer, USA) in the wavenumber range of 4000–400 cm^−1^. The thermal stability of the as-prepared materials was investigated over a temperature range of 30–650 °C, employing an acceleration rate of 5 °C·min^−1^ in an air stream, using a Q50 thermogravimetric analyzer (TA Instruments, New Castle, USA). The chemical states of Fe and O in the materials were examined using a K-Alpha photoelectron spectrometer (ThermoFisher Scientific, USA). The isotherms for N_2_ adsorption and desorption at 77 K over the materials were measured using a BELSORP-max apparatus (BEL, Japan). This allowed for calculating the surface area, using the Brunauer–Emmett–Teller (BET) model, and the total pore volume and pore size distribution, using the Horvath–Kawazoe (HK) model. The samples were activated under vacuum at 150 °C for 12 h before being introduced into the porosity analyzer.

### CO_2_ and N_2_ adsorption test

The characteristic adsorption of CO_2_ and N_2_ on the as-prepared materials was investigated in the range of 0–100 kPa at different temperatures using a static adsorption system (BELSORP-min II, BEL, Japan). Prior to implementation, approximately 250 mg of M-100Fe@Fe_2_O_3_ and MIL-100(Fe) samples were degassed under vacuum at 50 °C for 2 h, followed by an additional 12 h at 150 °C in a glass tube before naturally cooling to ambient temperature. To assess the adsorbent’s reusability, five cycles of CO_2_ adsorption and desorption were conducted at 25 °C. The adsorbent was refreshed for 2 h at 100 °C under vacuum for each cycle.

### Estimation of CO_2_/N_2_ selectivity via ideal adsorbed solution theory (IAST)

The IAST is widely recognized as a predictive means to evaluate the adsorption selectivity of an adsorbent towards a gas mixture without experimental data for the gas mixture. Herein, a binary mixture of CO_2_ and N_2_ containing 10 vol % CO_2_ is used to anticipate the CO_2_/N_2_ selectivity of the as-prepared materials. Initially, the experimental adsorption data for the single components CO_2_ and N_2_ were measured in a range of 0–100 kPa at 25 °C, which were then described using the Langmuir–Freundlich model [31–32]. The fitted parameters are given in Supporting Information File 1, Table S1 with high correlation coefficient values (*R*^2^ ≥ 0.9998) for all samples. Subsequently, the CO_2_/N_2_ selectivity over all samples was anticipated using IAST alongside the fitted parameters of the model. All Langmuir–Freundlich and IAST-CO_2_/N_2_ selectivity equations are detailed in Supporting Information File 1.

## Results and Discussion

### Material characterizations

**Figure 1:**
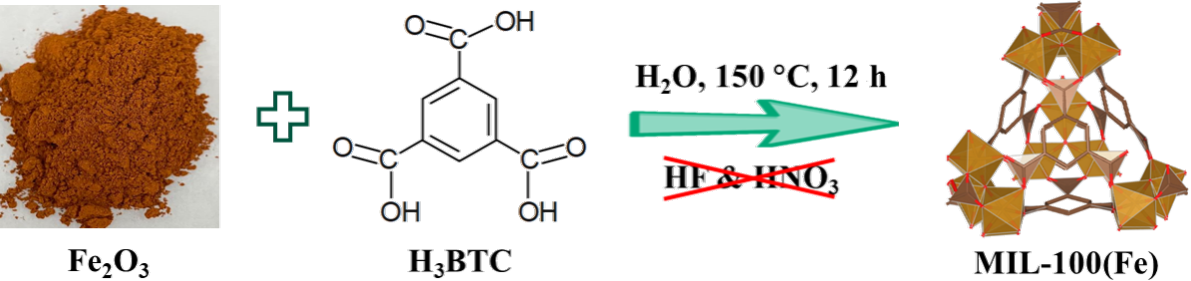
Pathway for preparing M-100Fe@Fe_2_O_3_ samples.

A range of M-100Fe@Fe_2_O_3_ samples was prepared from iron oxide (Fe_2_O_3_) nanoparticles as precursor with H_3_BTC as organic linker through a hydrothermal reaction as shown in Figure 1. The H_3_BTC concentration was incrementally introduced into the reaction system to control the quality of the as-prepared samples.

**Figure 2:**
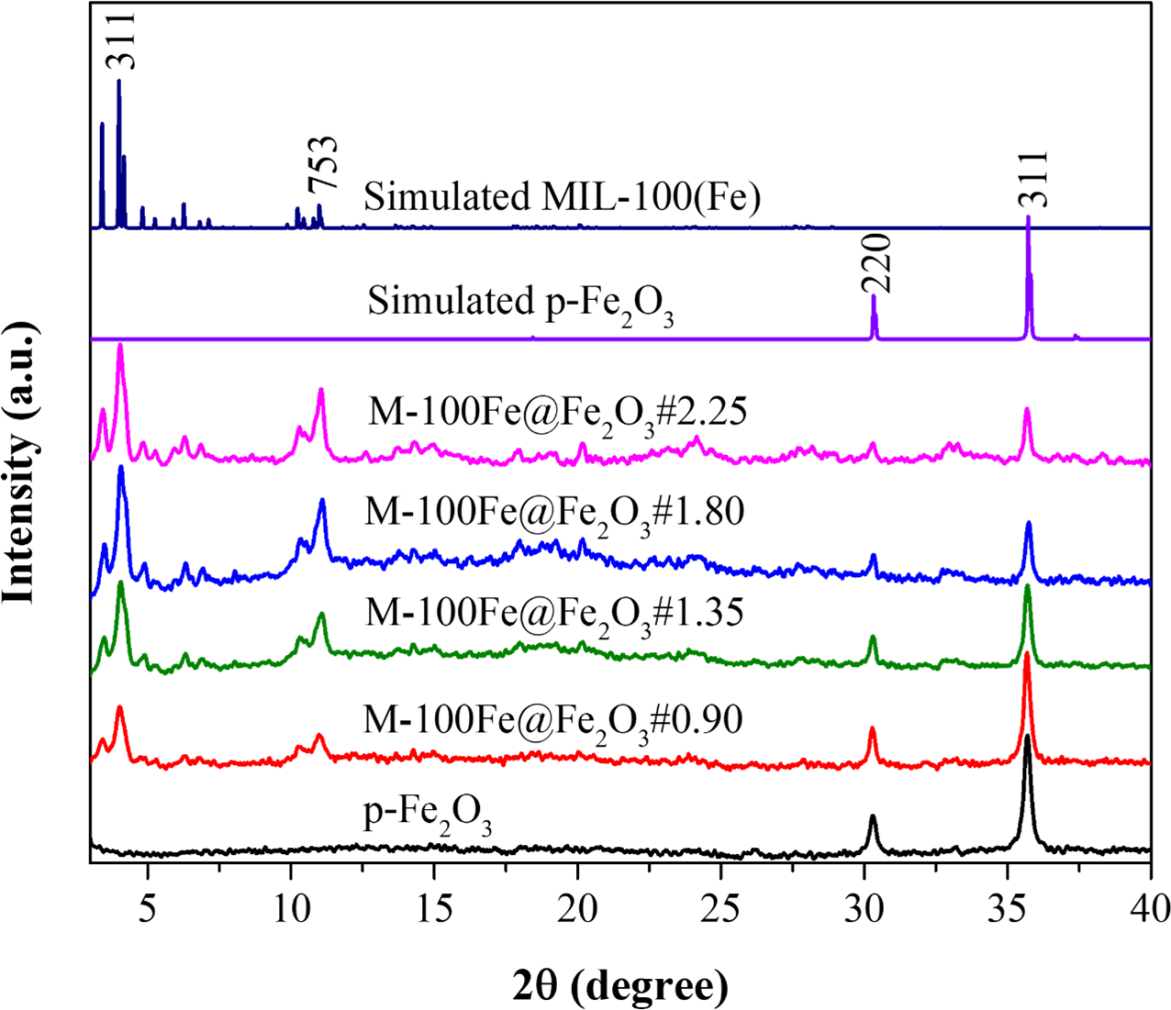
PXRD patterns of Fe_2_O_3_, MIL-100(Fe), and M-100Fe@Fe_2_O_3_ samples.

As shown in Figure 2, reflections of crystalline Fe_2_O_3_ were detected in the p-Fe_2_O_3_ XRD pattern, which are in absolute agreement with the simulated data, including the (220) and (311) planes in the examined region (JCPDS 39-1346). After the Fe_2_O_3_ nanoparticles underwent a highly conditional reaction, the characteristic peaks of MIL-100(Fe) were verified in the XRD profiles of the as-prepared M-100Fe@Fe_2_O_3_ samples. The XRD data of the reference MIL-100(Fe) sample and the simulated data from CCDC 640536 were aligned, demonstrating the successful preparation of MIL-100(Fe) crystals from Fe_2_O_3_ nanoparticles under the studied conditions. A further examination revealed that the measured peaks of MIL-100(Fe) gradually increased, whereas those of Fe_2_O_3_ decreased in intensity with increasing amounts of H_3_BTC from 0.9 to 1.80 g. However, even after increasing the H_3_BTC amount to 2.25 g in the reaction system, the characteristic peaks of MIL-100(Fe) and Fe_2_O_3_ were still preserved. This demonstrates that Fe_2_O_3_ nanoparticles were partially converted into MIL-100(Fe) material, with the quality of the obtained MIL-100(Fe) depending on the H_3_BTC concentration.

**Figure 3:**
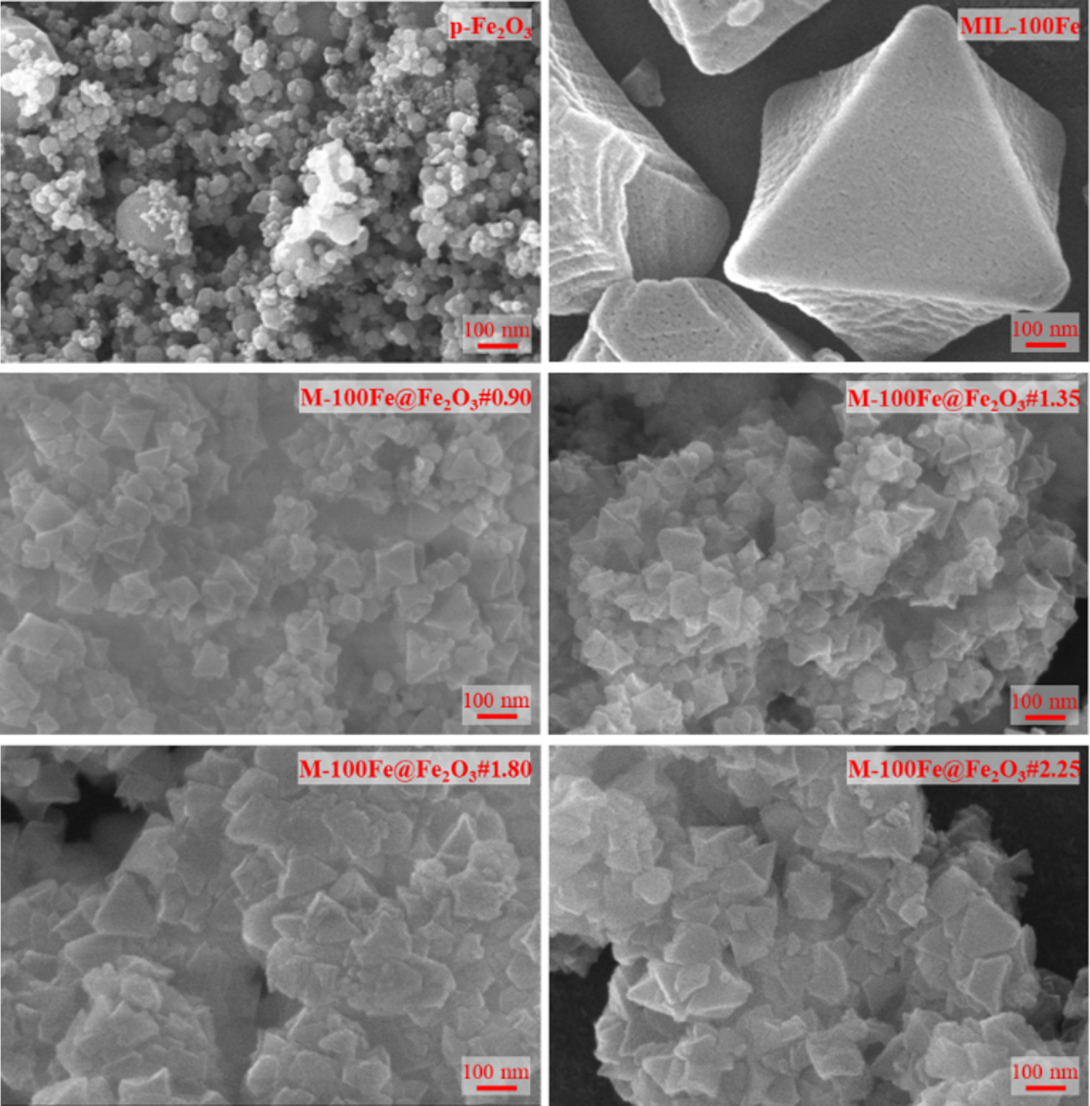
SEM images of Fe_2_O_3_, MIL-100(Fe), and M-100Fe@Fe_2_O_3_ samples.

To further reinforce this statement, the morphology of all materials was examined via SEM analysis. Figure 3 reveals that Fe_2_O_3_ nanoparticles appeared in spherical shapes, while MIL-100(Fe) crystals displayed an octahedral morphology with sizes around 1 μm. The SEM images of M-100Fe@Fe_2_O_3_ samples showed polyhedral nanoparticles, indicating the successful preparation of MIL-100(Fe) crystals. It is important to note that the morphology of the obtained MIL-100(Fe) crystals depends on both the source of Fe-based precursor and the synthetic approaches employed [24–25]. Additionally, the SEM images showed that spherical nanoparticles were easily observed in the M-100Fe@Fe_2_O_3_#0.90 and M-100Fe@Fe_2_O_3_#1.35 samples, whereas they were harder to detect in the M-100Fe@Fe_2_O_3_#1.80 and M-100Fe@Fe_2_O_3_#2.25 samples. This suggests that the concentration of MIL-100(Fe) crystals in the prepared M-100Fe@Fe_2_O_3_ samples increases in the following order: M-100Fe@Fe_2_O_3_#0.90 < M-100Fe@Fe_2_O_3_#1.35 < M-100Fe@Fe_2_O_3_#1.80 < M-100Fe@Fe_2_O_3_#2.25. This result aligns well with the XRD data shown in Figure 2.

**Figure 4:**
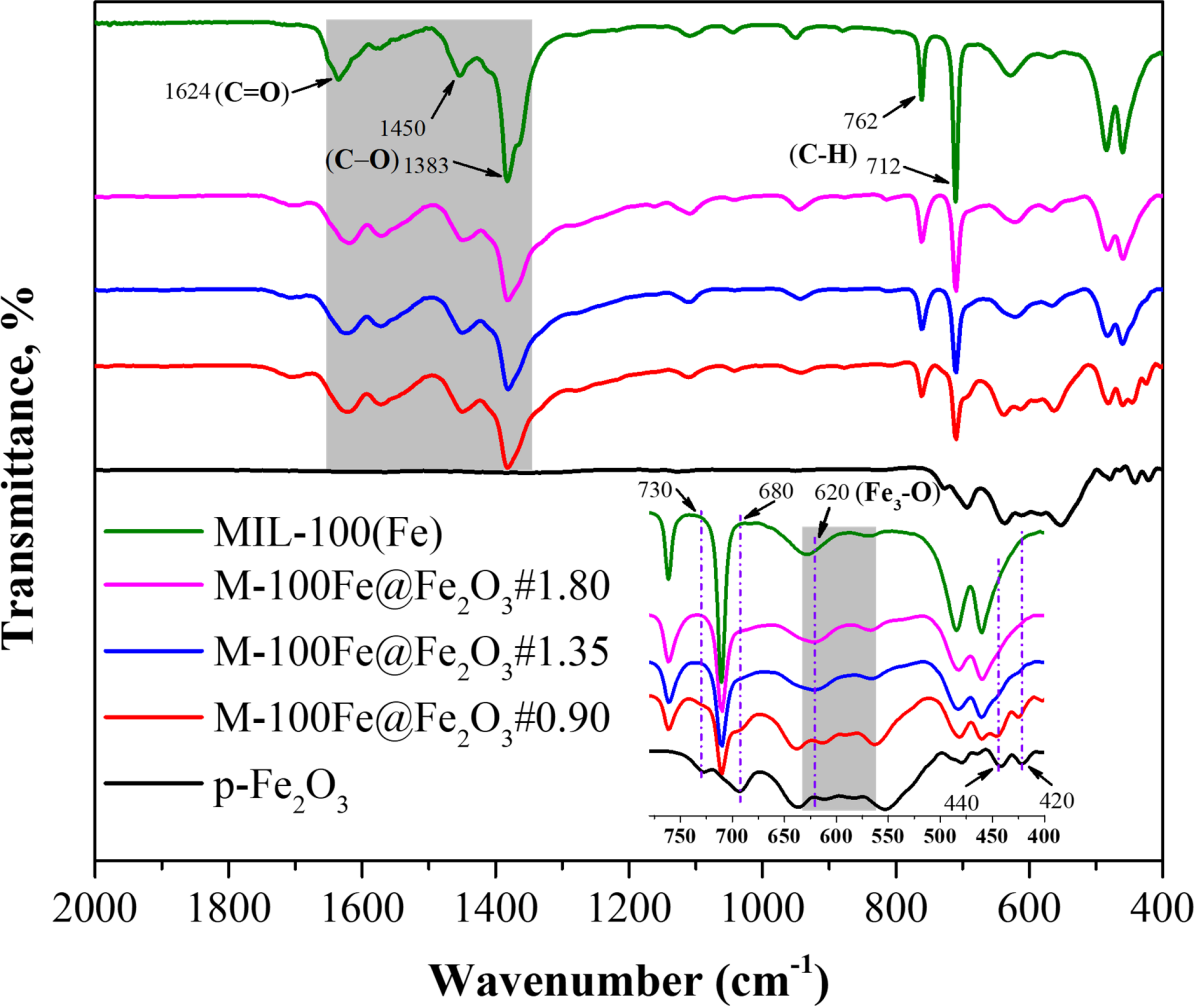
FTIR spectra of Fe_2_O_3_, MIL-100(Fe), and M-100Fe@Fe_2_O_3_ samples.

Figure 4 exhibits ATR-FTIR spectra of Fe_2_O_3_, MIL-100(Fe), and as-prepared M-100Fe@Fe_2_O_3_ samples. Characteristic peaks, including vibration bands in the region of 550 to 630 cm^−1^, were assigned to Fe–O bonds in the Fe_2_O_3_ structure [33], and a signal at 620 cm^−1^ was attributed to the vibration of Fe(III)–O bonds in oxo-centered trinuclear iron complexes (Fe_3_–O) within the MIL-100(Fe) framework [32]. Besides, the vibrations at 712 and 760 cm^−1^ as well as in the region of 1380–1620 cm^−1^, which were only detected on the FTIR spectra of M-100(Fe)@Fe_2_O_3_ samples, are well known as characteristic signals of MIL-100(Fe) [9,32]. It is noteworthy that the distinctive signals of MIL-100(Fe) displayed increasing intensity, while the signals associated with Fe_2_O_3_ (specifically at 420, 440, 680, and 730 cm^−1^, as depicted in Figure 4) gradually weakened as the H_3_BTC content was elevated from 0.90 to 1.80 g. These combined observations provide strong evidence of the successful synthesis of MIL-100(Fe) from Fe_2_O_3_ nanoparticles; the proportion of MIL-100(Fe) in the final material depends on the quantity of H_3_BTC introduced into the reaction system.

**Figure 5:**
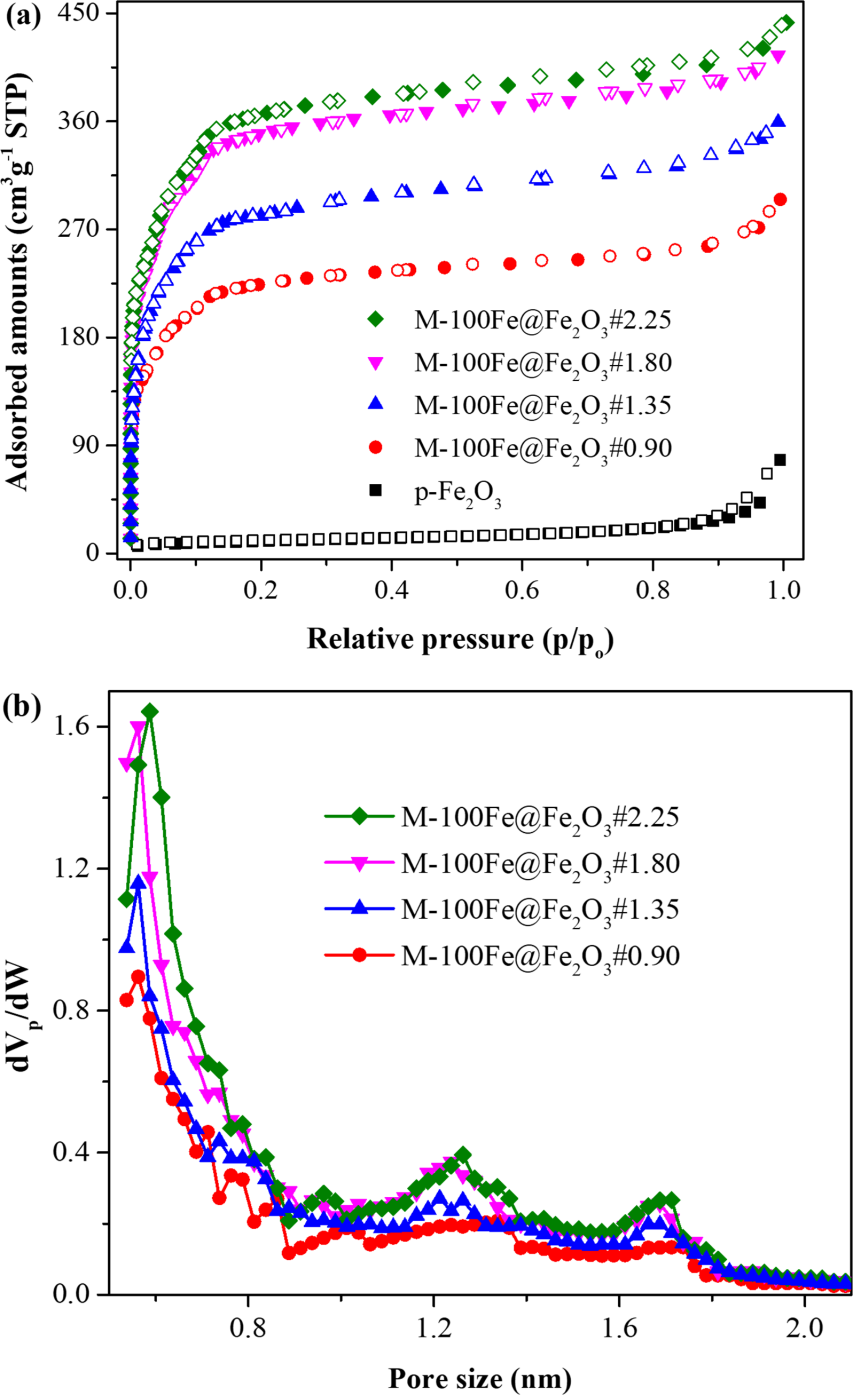
(a) N_2_ adsorption and desorption isotherms over Fe_2_O_3_, MIL-100(Fe), and M-100Fe@Fe_2_O_3_ samples obtained at 77 K. (b) Pore size distribution of M-100Fe@Fe_2_O_3_ and reference MIL-100(Fe) samples calculated using the HK model.

The textural properties of all materials were determined through analysis of N_2_ adsorption and desorption isotherms. Figure 5a illustrates a dense structure for Fe_2_O_3_ nanoparticles, with isotherms exhibiting a type-III profile according to IUPAC. It reflects a limited BET surface area and a pore volume of 36.3 m^2^·g^−1^ and 0.118 cm^3^·g^−1^, respectively (Table 1). In contrast, the reference MIL-100(Fe) sample exhibited N_2_ adsorption/desorption isotherms characteristic of type I. Additionally, a pore size distribution was observed with three prominent peaks at approximately 0.62, 1.30, and 1.75 nm within the micropore region defined by IUPAC (less than 2.0 nm), as depicted in Figure 5b. These observations confirm that the reference sample has a microporous structure with a BET surface area and a total pore volume of 1825.4 m^2^·g^−1^ and 0.772 cm^3^·g^−1^, respectively. Similarly, the micropore structures were also determined in the obtained M-100Fe@Fe_2_O_3_ samples by examining the profiles of isotherms and pore size distributions. Notably, the adsorbed amount of N_2_ (Figure 5a) and the pore volume of the three primary peaks (Figure 5b) exhibited a gradual increase from M-100Fe@Fe_2_O_3_#0.90 to M-100Fe@Fe_2_O_3_#2.25 samples. The BET surface area and the total pore volume reached 858.9 m^2^·g^−1^ and 0.451 cm^3^·g^−1^ for M-100Fe@Fe_2_O_3_#0.90, respectively. Subsequently increasing the H_3_BTC amount up to 1.80 g caused an improvement in BET surface area to 1365.4 m^2^·g^−1^ and total pore volume to 0.642 cm^3^·g^−1^ for M-100Fe@Fe_2_O_3_#1.80. There was a further slight enhancement in the porosity of the obtained material when increasing the H_3_BTC amount from 1.80 to 2.25 g, corresponding to 1433.8 m^2^·g^−1^ and 0.671 cm^3^·g^−1^ for BET surface area and total pore volume of M-100Fe@Fe_2_O_3_#2.25, respectively. Considering the trade-off between required H_3_BTC amount and porosity of the obtained materials and within the scope of our investigation, M-100Fe@Fe_2_O_3_#1.8 stands out as the optimal sample, exhibiting a BET surface area of 1365.4 m^2^·g^−1^, which surpasses the values of 730 m^2^·g^−1^ [26] and 1244.6 m^2^·g^−1^ [27] observed in MIL-100(Fe) samples prepared from Fe_3_O_4_.

**Table 1:** Textural properties of Fe_2_O_3_ and the as-prepared samples.

Samples	BET surface area (m^2^·g^−1^)	Total pore volume (cm^3^·g^−1^)

p-Fe_2_O_3_	36.3	0.118
M-100Fe@Fe_2_O_3_#0.90	858.9	0.451
M-100Fe@Fe_2_O_3_#1.35	1065.1	0.526
M-100Fe@Fe_2_O_3_#1.80	1365.4	0.642
M-100Fe@Fe_2_O_3_#2.25	1433.8	0.671
MIL-100(Fe)	1825.4	0.772

**Figure 6:**
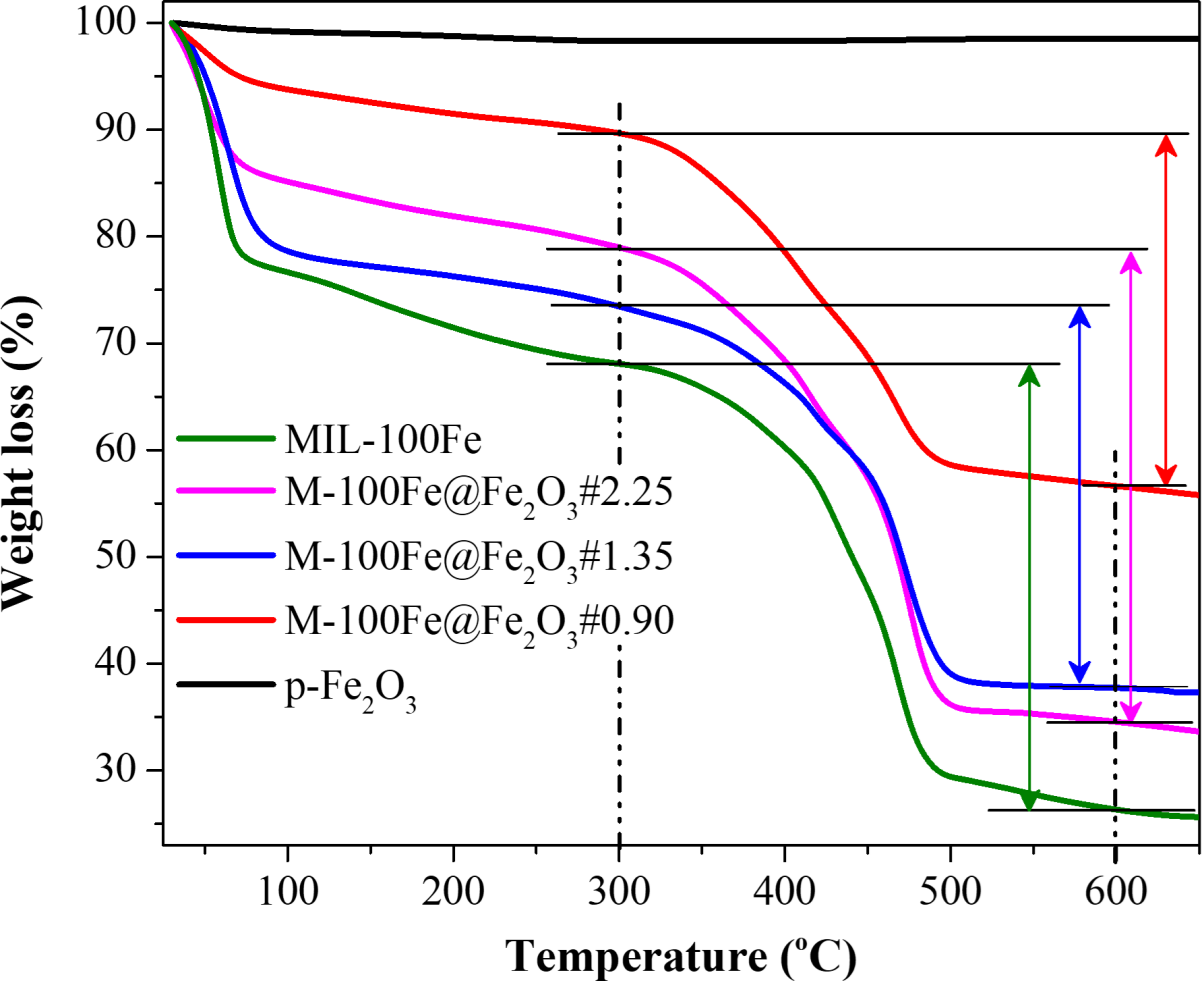
TGA curves of Fe_2_O_3_, MIL-100(Fe), and M-100Fe@Fe_2_O_3_ samples.

Thermogravimetric analyses were performed on Fe_2_O_3_ and M-100Fe@Fe_2_O_3_ samples to confirm the presence of the MIL-100(Fe) phase in the resulting materials and to estimate the conversion of Fe_2_O_3_ into MIL-100(Fe) material. The TGA profile of Fe_2_O_3_ (Figure 6) showed only a very small weight loss in the investigated temperature range due to a small quantity of absorbed moisture in the sample. Contrarily, the TGA profiles of M-100Fe@Fe_2_O_3_#0.90, M-100Fe@Fe_2_O_3_#1.35, and M-100Fe@Fe_2_O_3_#1.80 samples revealed similar mechanisms in weight loss over a temperature range of 30 to 650 °C, coinciding with three stages of weight loss observed in the reference sample MIL-100(Fe). Initially, the trapped molecules (ethanol and water) inside the pore system were released at low temperature around 100 °C. Subsequently, the ligands (water and/or –OH ligands) connected to Fe sites of the iron oxo-clusters were removed, leaving unsaturated metal sites inside the framework. Finally, a significant weight loss (approx. 41.8%) occurred due to the decomposition of the organic linkers in the framework at high temperatures between 300 and 650 °C. The process continued until the sample’s mass stabilized at 26.3 wt % of Fe_2_O_3_ at 600 °C [9,11,34]. As a result, the mass percentage of MIL-100(Fe) was calculated to be 65.5% based on the formula of MIL-100(Fe) (Fe_3_O[C_6_H_3_(COO)_3_]_2_) after the removal of –OH and H_2_O at 300 °C. This result matches with the value of 68.1% attained from the TGA profile of MIL-100(Fe) at 300 °C. Similarly, at 600 °C, the mass percentage of Fe_2_O_3_ reached 56.6%, 37.7%, and 34.5% for the M-100Fe@Fe_2_O_3_#0.90, M-100Fe@Fe_2_O_3_#1.35, and M-100Fe@Fe_2_O_3_#1.80 samples, respectively (Figure 6). As discussed, Fe_2_O_3_ was only partially converted into MIL-100(Fe); hence, the obtained Fe_2_O_3_ from TGA curves included both Fe_2_O_3_ derived from M-100(Fe)@Fe_2_O_3_ and the original Fe_2_O_3_ reactant. Combining the obtained data from the reference sample MIL-100(Fe), the mass percentages of Fe_2_O_3_ reactant were inferred to be 35.8%, 15.2%, and 6.6%, corresponding to conversion rates of 36.7%, 59.6%, and 81% for M-100Fe@Fe_2_O_3_#0.90, M-100Fe@Fe_2_O_3_#1.35, and M-100Fe@Fe_2_O_3_#1.80 samples, respectively.

**Figure 7:**
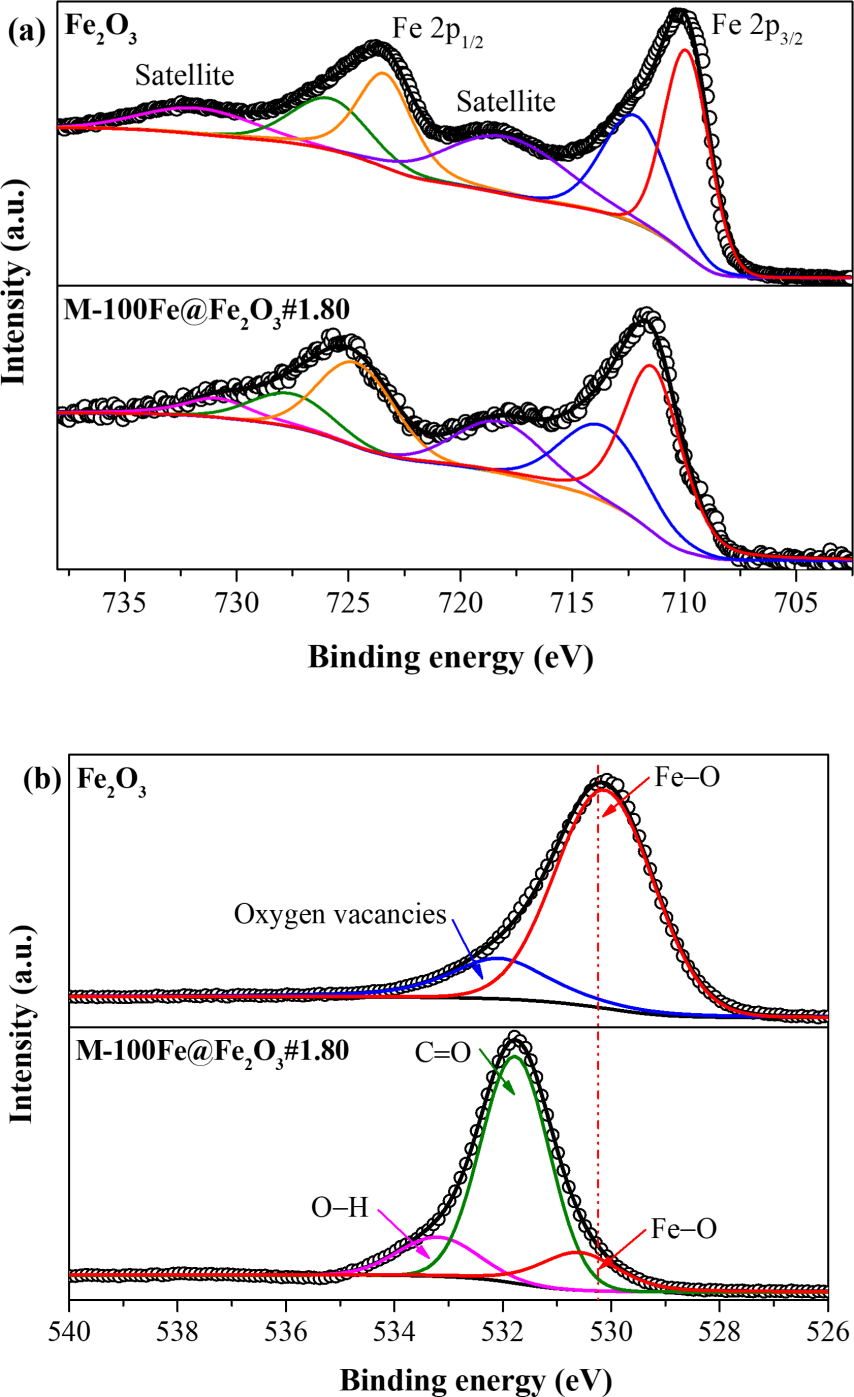
XPS spectra of Fe_2_O_3_ and M-100Fe@Fe_2_O_3_#1.80 samples: (a) Fe 2p region and (b) O 1 s region.

Figure 7 displays the oxidation states of Fe and O in both Fe_2_O_3_ and M-100Fe@Fe_2_O_3_#1.80 samples. In both cases, the Fe 2p spectra feature two prominent peaks at approximately 710 and 725 eV, accompanied by two shoulder peaks at around 718 and 731 eV. These peaks were attributed to Fe(III), corresponding to the Fe 2p_3/2_ and Fe 2p_1/2_ states, respectively [24,32]. Nevertheless, a substantial difference regarding the oxygen constituents was observed when examining the deconvoluted spectra of O 1s in Fe_2_O_3_ and M-100Fe@Fe_2_O_3_#1.80 samples. As shown in Figure 7b, the O 1s spectrum of the Fe_2_O_3_ sample exhibits two peaks, that is, a major peak at 530.1 eV standing for Fe–O bonds and a minor peak at 532 eV assigned to oxygen-related vacancies within the framework of Fe_2_O_3_ [35]. In contrast, the O 1s spectrum of the M-100Fe@Fe_2_O_3_#1.80 sample was composed of three peaks, namely, a major peak at 531.6 eV for C=O bonds and two minor peaks, one at 530.1 eV for Fe–O bonds and the other at 533.5 eV for O–H bonds [32,36]. It is noteworthy that Fe–O predominates in the Fe_2_O_3_ structure, whereas in the M-100Fe@Fe_2_O_3_#1.80 sample, the C=O is predominant. This difference is due to the presence of O atoms in the M-100Fe@Fe_2_O_3_#1.80 sample, which primarily correspond to bridges of benzene-1,3,5-tricarboxylate in the MIL-100(Fe) framework.

### CO_2_/N_2_ separation performance

**Figure 8:**
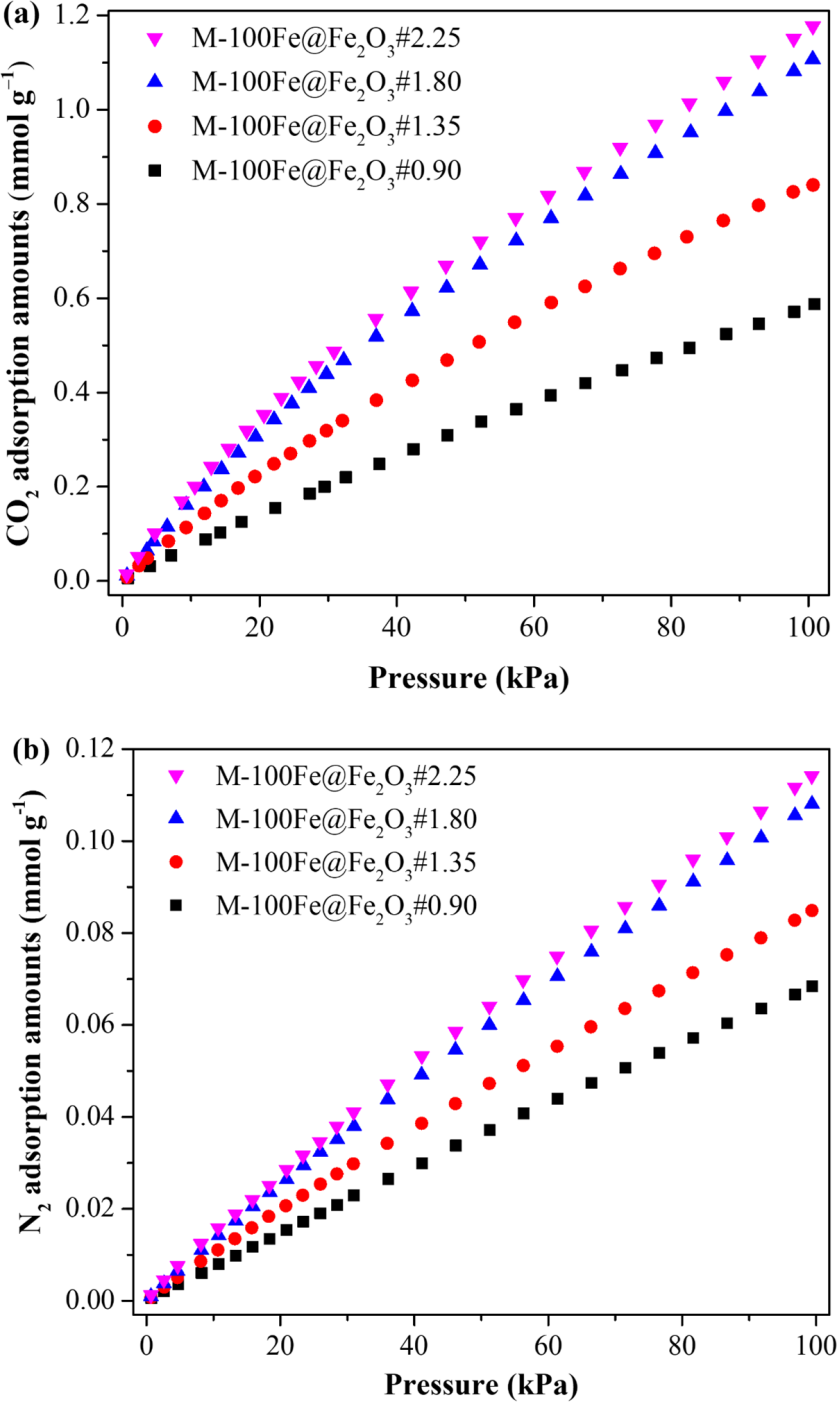
Adsorption isotherms of M-100Fe@Fe_2_O_3_ and reference MIL-100(Fe) samples at 298 K: (a) CO_2_ adsorption isotherms, (b) N_2_ adsorption isotherms.

**Figure 9:**
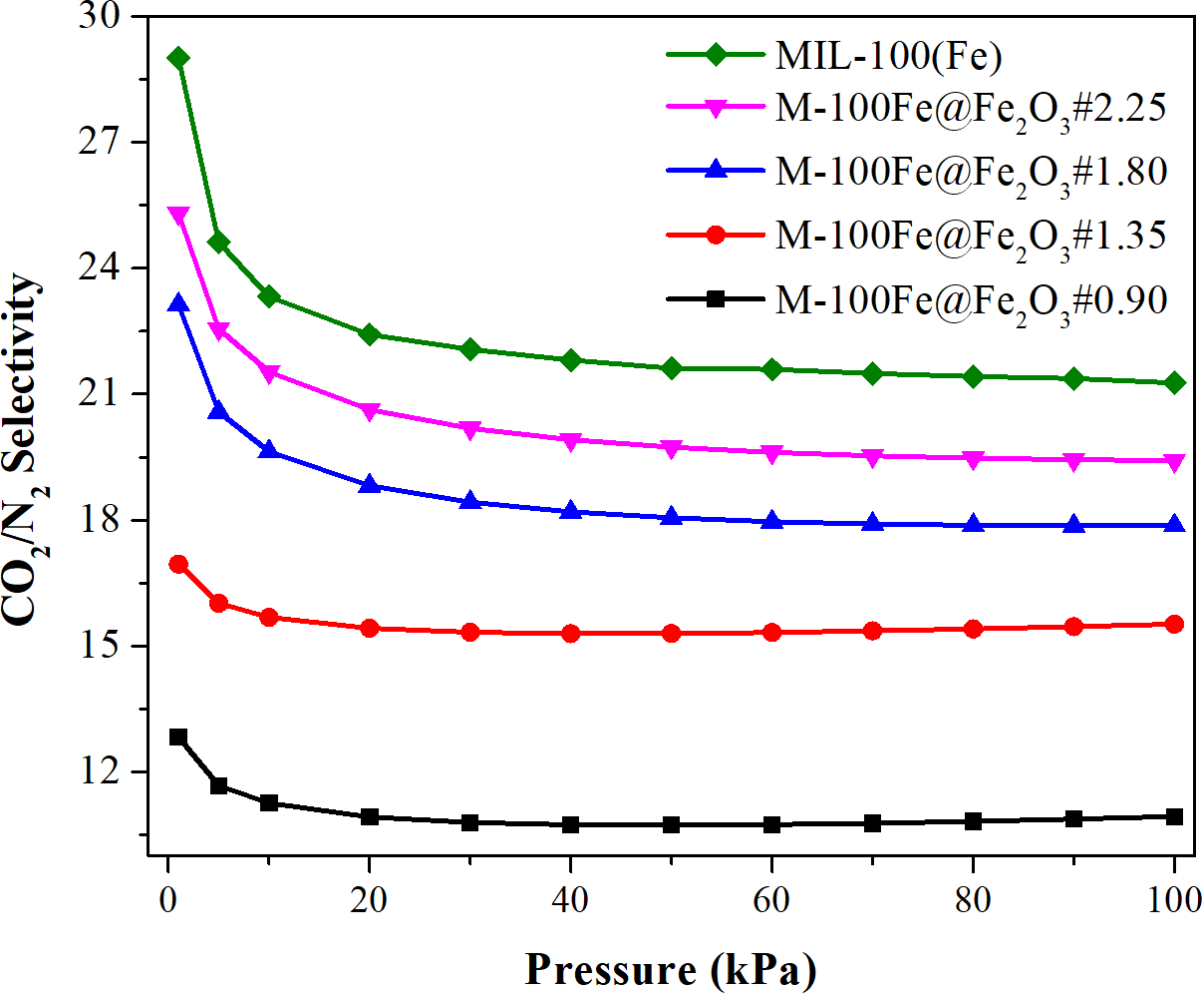
IAST-derived CO_2_/N_2_ selectivities over M-100Fe@Fe_2_O_3_ samples for a mixture with 10% N_2_ and 90% CO_2_ at 298 K.

To evaluate the effectiveness of CO_2_/N_2_ separation in the as-prepared samples, the adsorption behavior of CO_2_ and N_2_ was investigated at pressures ranging from 0 to 100 kPa at 298 K. As exhibited in Figure 8, a series of the as-prepared samples was arranged in the order of M-100Fe@Fe_2_O_3_#0.90 < M-100Fe@Fe_2_O_3_#1.35 < M-100Fe@Fe_2_O_3_#1.80 < M-100Fe@Fe_2_O_3_#2.25 < MIL-100(Fe) (reference sample) with a gradual increment in adsorption capacity for both CO_2_ and N_2_ due to the increasing BET surface area (Table 1). It is well known that the MIL-100(Fe) framework contains an abundance of active sites, including unsaturated metal sites, and –OH and free –COOH groups, which perhaps form adsorptive interactions with CO_2_ molecules [32]. Consequently, the samples showed outstanding CO_2_ uptake in comparison with N_2_. Additionally, in Figure 9, the CO_2_/N_2_ separation performance of samples is depicted through IAST prediction of the CO_2_/N_2_ adsorptive selectivity (Supporting Information File 1). These predictions are based on a gas mixture containing 10% CO_2_ and 90% N_2_ by volume at 298 K. As displayed in Figure 9, all samples showed high potential for CO_2_/N_2_ separation at low pressure; at higher pressures, the separation performance gradually declined and reached plateau values. However, at the same bulk pressure, the adsorptive selectivity for CO_2_ over N_2_ of the adsorbents increased gradually because of the rising BET surface area resulting from an increase in the H_3_BTC amount in the reaction system from 0.90 to 2.25 g. A similar trend was observed in the porosity analysis when increasing the H_3_BTC amount from 1.80 to 2.25 g. It corresponds to a negligible improvement in CO_2_ uptake, changing from 1.10 to 1.18 mmol·g^−1^ at 25 °C and 100 kPa. Taking into account the balance between CO_2_ adsorption performance and the quantity of H_3_BTC consumed, M-100Fe@Fe_2_O_3_#1.80 stands out as the preferred sample, exhibiting a CO_2_ uptake capacity of 1.1 mmol·g^−1^ and a CO_2_/N_2_ selectivity of 18 at 25 °C and 100 kPa. These findings have been compared with the CO_2_ uptake capacities of MIL-100(Fe)-based adsorbents created through different methods, as well as other MOFs and conventional materials, all of which are detailed in Table S2 (Supporting Information File 1).

### Isosteric enthalpy of CO_2_ adsorption over M-100Fe@Fe_2_O_3_#1.80 and its reusability

**Figure 10:**
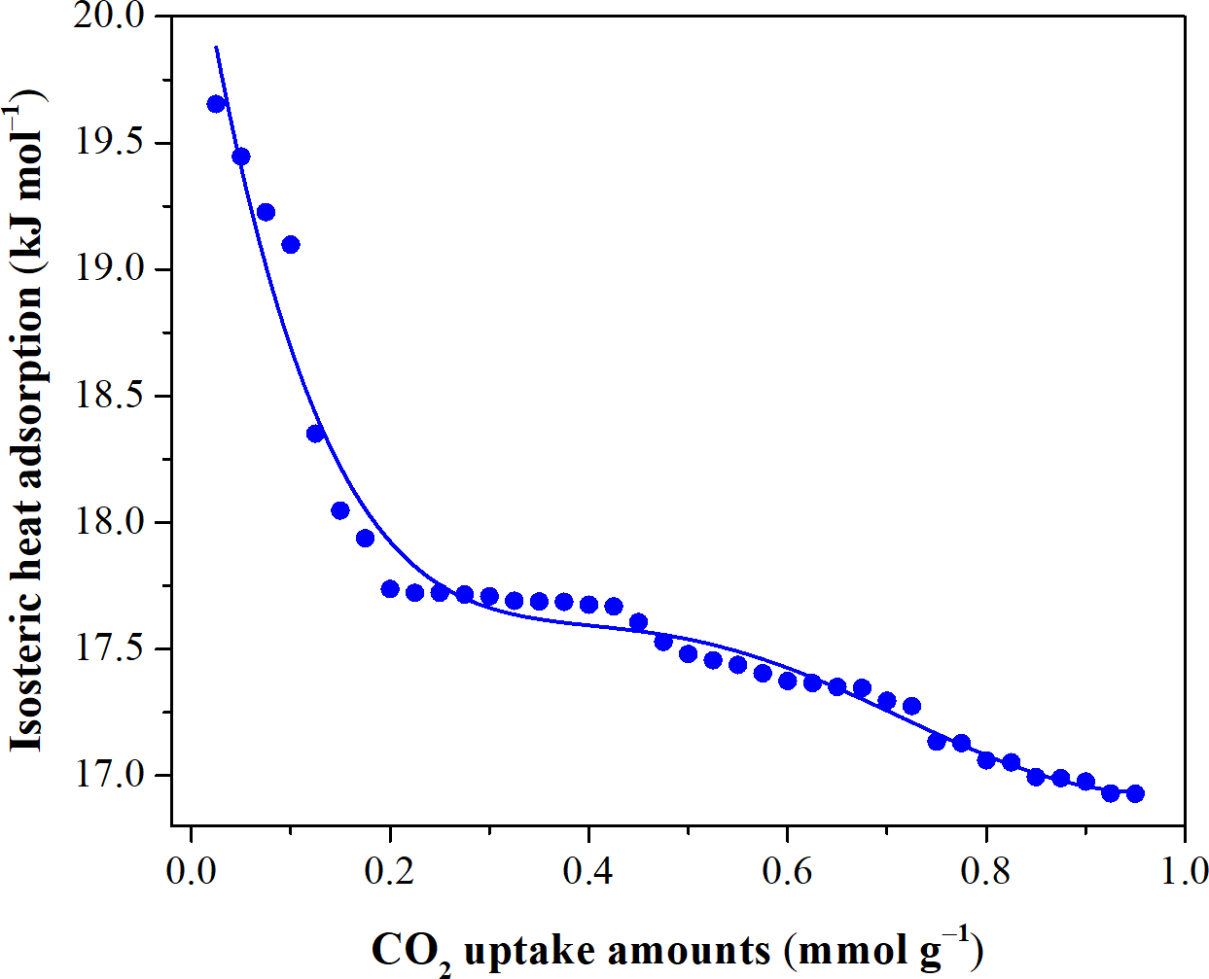
Enthalpy of CO_2_ adsorption over M-100Fe@Fe_2_O_3_#1.80 sample.

**Figure 11:**
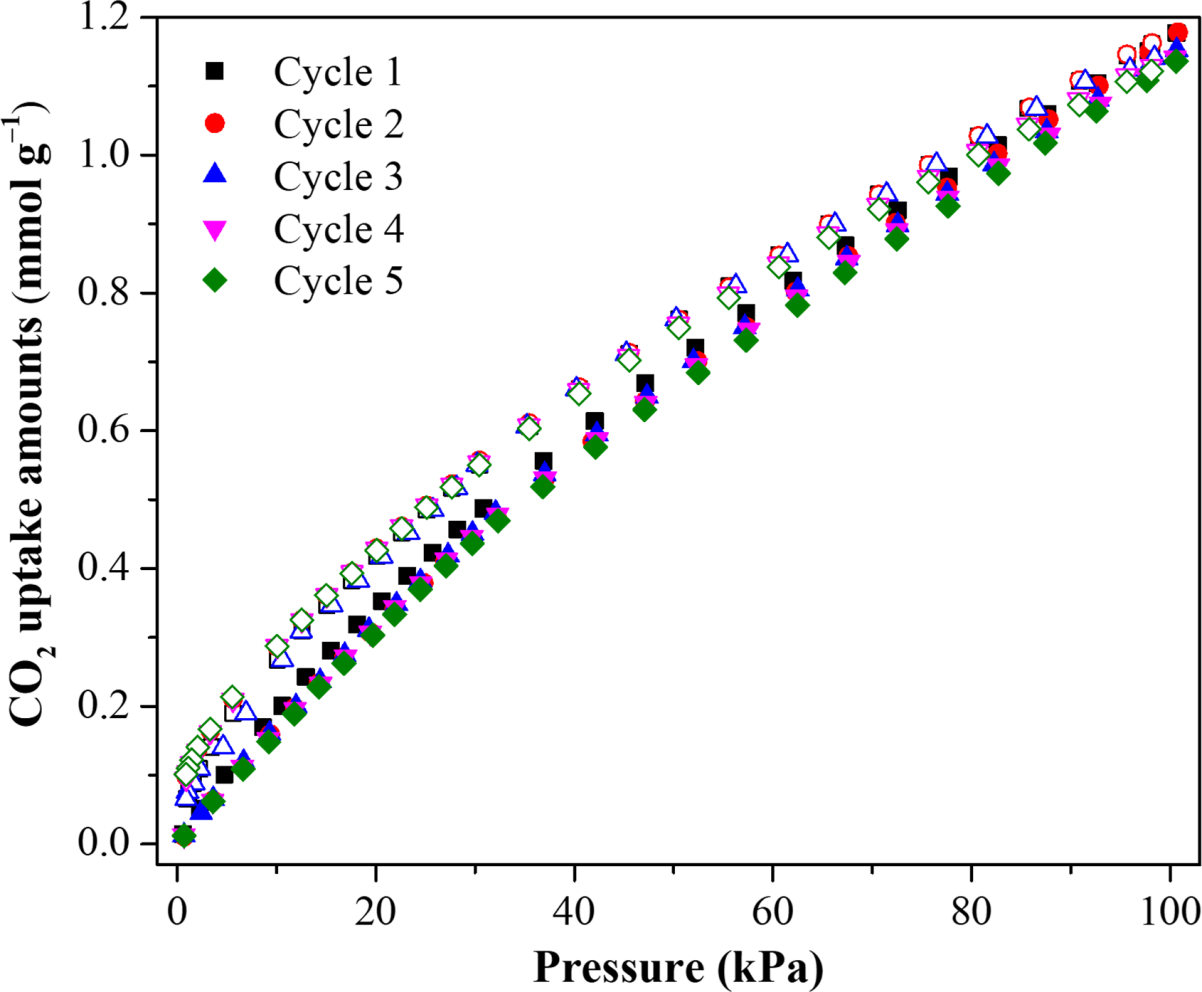
CO_2_ adsorption/desorption isotherms over M-100Fe@Fe_2_O_3_#1.80 sample for five cycles at 298 K.

To explore the inherent relationship between the target gas and the adsorbent, the adsorption behavior of CO_2_ over the M-100Fe@Fe_2_O_3_#1.80 sample was studied in a temperature range from 5 to 35 °C. As observed in the isotherms in Figure S1 (Supporting Information File 1), the CO_2_ uptake was proportional to the pressure and inversely proportional to temperature, suggesting that adsorptive interactions based on physical bonds took place between CO_2_ molecules and M-100Fe@Fe_2_O_3_#1.80 [31,37]. To support this assertion, the Clausius–Clayperon equation was employed to determine the isosteric enthalpy of CO_2_ adsorption (Supporting Information File 1). As anticipated, the capture of CO_2_ molecules using M-100Fe@Fe_2_O_3_#1.80 releases approximately 20 to 17 kJ·mol^−1^ when the CO_2_ uptake is increased up to 1.0 mmol·g^−1^ (Figure 10). The obtained results fall within the value range defined for the physisorption regime [37–38], implying that a spent M-100Fe@Fe_2_O_3_#1.80 sample could be easily refreshed under soft conditions for reuse, thus, extending the material’s life cycle. To affirm this aspect, a conservative trial of five cycles for CO_2_ adsorption/desorption was carried out at 298 K as shown in Figure 11. The CO_2_ uptake over the M-100Fe@Fe_2_O_3_#1.80 sample was maintained in comparison with the fresh sample after the five cycles.

## Conclusion

For the first time, a facile synthetic method was employed to effectively fabricate MIL-100(Fe) from Fe_2_O_3_ using a typical hydrothermal process. By adjusting the mass ratio between Fe_2_O_3_ and H_3_BTC in the reaction system, a continuous enhancement in the quality of the produced MIL-100(Fe) was achieved. The optimal conditions for producing MIL-100(Fe) material were determined to be 150 °C for 12 h, with a mass ratio of 0.9/1.80 (g/g) for Fe_2_O_3_/H_3_BTC, as a trade-off between required H_3_BTC amount and BET surface area. This resulted in an 81% conversion and a BET surface area of 1365.4 m^2^·g^−1^ for the M-100Fe@Fe_2_O_3_#1.80 sample. Furthermore, M-100Fe@Fe_2_O_3_#1.80 demonstrated promising potential as an adsorbent for CO_2_/N_2_ separation, exhibiting a high CO_2_ uptake of 1.1 mmol·g^−1^ and a CO_2_/N_2_ selectivity of 18 at 25 °C and 100 kPa. Importantly, it retained a significant CO_2_ adsorption capacity even after five reuse cycles. The use of Fe_2_O_3_ in conjunction with an ecologically benign technology offers a new pathway for producing MIL-100(Fe), potentially utilizing a cheap and readily accessible raw material source from the iron and steel industry, thereby reducing production cost.

## Supporting Information

Procedure to preparation of reference MIL-100(Fe). Langmuir–Freundlich model and the calculated IAST-CO_2_/N_2_ selectivity equations. The Clausius–Clayperon equation for calculating CO_2_ heat adsorption. The characteristic parameters alongside correlation coefficients from fitting the Langmuir–Freundlich model (Table S1). CO_2_ uptake capacity on various adsorbents at 1 bar (Table S2). CO_2_ isotherms at several different temperature on M-100(Fe)@Fe_2_O_3_#1.80 sample (Figure S1). EDX mapping of elemental Fe and C on the M-100Fe@Fe_2_O_3_#1.80 sample (Figure S2).

File 1 Additional experimental data.

## Data Availability

The data that supports the findings of this study is available from the corresponding author upon reasonable request.
