# Supplementary material for: Facile synthesis of Fe-based metal–organic frameworks from Fe2O3 nanoparticles and their application for CO2/N2 separation
Source: Beilstein J Nanotechnol. 2024 Jul 19;15:897–908. doi: 10.3762/bjnano.15.74 (PMC11285048; doi:10.3762/bjnano.15.74)
Supplement: File 1 — Additional experimental data. [file Beilstein_J_Nanotechnol-15-897-s001.pdf]

BEILSTEIN JOURNAL OF NANOTECHNOLOGY

# Supporting Information

for

# Facile synthesis of Fe-based metal–organic frameworks from $Fe_2O_3$ nanoparticles and their application for $CO_2/N_2$ separation

Van Nhieu Le, Hoai Duc Tran, Minh Tien Nguyen, Hai Bang Truong, Toan Minh Pham and Jinsoo Kim

*Beilstein J. Nanotechnol.* **2024,** *15,* 897–908. doi:10.3762/bjnano.15.74

## Additional experimental data

License and Terms: This is a supporting information file under the terms of the Creative Commons Attribution License (https://creativecommons.org/licenses/by/4.0). Please note that the reuse, redistribution and reproduction in particular requires that the author(s) and source are credited and that individual graphics may be subject to special legal provisions.
The license is subject to the *Beilstein Journal of Nanotechnology* terms and conditions: (https://www.beilstein-journals.org/bjnano/terms)

## Preparation of the reference MIL-100(Fe) sample

The reaction mixture was prepared with a molar composition of 1.0 Fe: 0.67 $H_3BTC$: 1.2 $HNO_3$: 2.0 HF: 280 $H_2O$ in a 100 mL Teflon-lined autoclave. It was stirred for 20 min at ambient temperature. Subsequently, the mixture was tightly sealed and incubated at 160 °C for 24 h in an electric oven. Afterward, it was cooled down to room temperature. The MIL-100(Fe) was then retrieved from the orange slurry after centrifugation and subjected to multiple washes with dimethylformamide and ethanol to eliminate any residual reactants from the product mixture. Finally, the MIL-100(Fe) was left to dry overnight at 70 °C.

**Table S1:** The characteristic parameters alongside correlation coefficients from fitting the Langmuir–Freundlich (L–F) model.

| Samples | The obtained parameters from fitting $CO_2$ | | | |
|---|---|---|---|---|
| | $q_{sat}$ (mmol·g$^{-1}$) | $a$ (kPa$^{-1}$) | $n$ | $R^2$ |
| M-100Fe@$Fe_2O_3$#0.90 | 3.59748 | 0.00225 | 0.96613 | 0.9999 |
| M-100Fe@$Fe_2O_3$#1.35 | 4.47178 | 0.0035 | 0.91115 | 0.9999 |
| M-100Fe@$Fe_2O_3$#1.80 | 5.75658 | 0.00417 | 0.87543 | 0.9998 |
| M-100Fe@$Fe_2O_3$#2.25 | 6.31350 | 0.00446 | 0.8521 | 0.9998 |
| MIL-100(Fe) | 5.29325 | 0.00873 | 0.86948 | 0.9998 |

| Samples | The obtained parameters from fitting $N_2$ | | | |
|---|---|---|---|---|
| | $q_{sat}$ (mmol·g$^{-1}$) | $a$ (kPa$^{-1}$) | $n$ | $R^2$ |
| M-100Fe@$Fe_2O_3$#0.90 | 0.48828 | 0.00143 | 1.02837 | 0.9998 |
| M-100Fe@$Fe_2O_3$#1.35 | 0.97068 | 0.00123 | 0.94648 | 0.9999 |
| M-100Fe@$Fe_2O_3$#1.80 | 1.28516 | 0.00119 | 0.94557 | 0.9999 |
| M-100Fe@$Fe_2O_3$#2.25 | 1.60773 | 0.00111 | 0.91890 | 0.9999 |
| MIL-100(Fe) | 0.65055 | 0.00372 | 0.97849 | 0.9998 |

The Langmuir–Freundlich (L–F) model is described as the following equation [1,2]:

$$q = q_{\mathrm{sat}} \frac{ap^n}{1+ap^n} \qquad (1)$$

Where $q$ (mmol·g$^{-1}$) stands for the uptake amount; $q_{sat}$ (mmol·g$^{-1}$) stands for the saturation uptake capacity on the adsorptive sites; $p$ (kPa) is the pressure of bulk gas at equilibrium with the adsorbed phase; $a$ (kPa$^{-1}$) is the affinity coefficient of the sites; $n$ is the coefficient of the L–F model.

The $CO_2/N_2$ selectivity was calculated using the formula as below [3]:

$$S_{CO_2/N_2} = \frac{q_{CO2}/p_{CO2}}{q_{N2}/p_{N2}} \quad (2)$$

Where $q_{CO2}$ and $q_{N2}$ are loading amounts of $CO_2$ and $N_2$ in mmol $g^{-1}$ estimated by IAST, respectively. $p_{CO2}$ and $P_{N2}$ are the partial pressure of $CO_2$ and $N_2$ in kPa, respectively.

The Clausius–Clayperon equation is described as the following equation [1,4]:

$$lnP = -\frac{Q_{st}}{RT} + C \quad (3)$$

Herein, $P$ is the pressure in kPa, $R$ is the ideal gas constant in J $mol^{-1}$ $K^{-1}$, $T$ is the absolute temperature in K, and $C$ is the constant in the dimensionless unit.

**Table S2:** Compared $CO_2$ uptake capacity on various adsorbents at 1 bar.

| Adsorbents | Adsorption capacity ($mmol \cdot g^{-1}$) | Temperature (K) | References |
|---|---|---|---|
| ZSM-5 | 1.50 | 303 | [5] |
| MCM-41 | 0.79 | 298 | [6] |
| Mesoporous alumina | 1.18 | 298 | [7] |
| SBA-15 | 0.47 | | |
| γ-Alumina | 0.68 | | |
| Zeolite-13X | 1.70 | 298 | [8] |
| ZIF-8 | 0.83 | | |
| ZIF-70 | 1.43 | 298 | [9] |
| UIO-66 | 1.79 | 298 | [10] |
| d-UiO-6-2.0HCl | 1.42 | 298 | [11] |
| UiO-66_FA_mod | 1.44 | 298 | [12] |
| MF-MOF-801 | 1.37 | 298 | [13] |
| MF-MOF-804 | 1.00 | | |
| MW-MIL-100(Fe) | 1.50 (1981 $m^2 \cdot g^{-1\#}$; 250 °C♣) | 298 | [14] |
| MIL-100(Fe) | 1.29 (1107 $m^2 \cdot g^{-1\#}$; 120 °C♣) | 298 | [15] |
| MIL-100(Fe) | 0.92 (1083 $m^2 \cdot g^{-1\#}$; 150 °C♣) | 298 | [16] |
| MIL-100(Fe)<br>M-100Fe@$Fe_2O_3$#1.80 | 1.77 (1825.4 $m^2 \cdot g^{-1\#}$; 150 °C♣)<br>1.11 (1365.4 $m^2 \cdot g^{-1\#}$; 150 °C♣) | 298 | This work |

# BET surface area; ♣Activation temperature before measuring $CO_2$ adsorption capacity over the absorbents

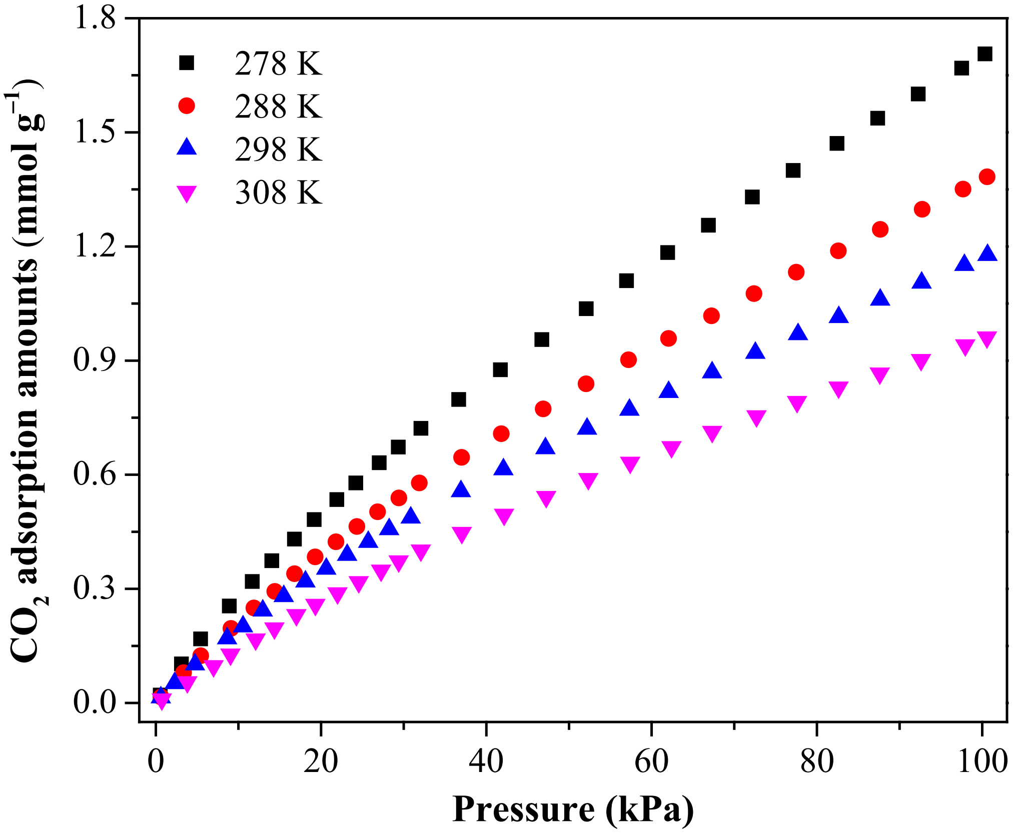


**Figure S1:** $CO_2$ isotherms at several different temperatures on M-100(Fe)@$Fe_2O_3$#1.80 sample.

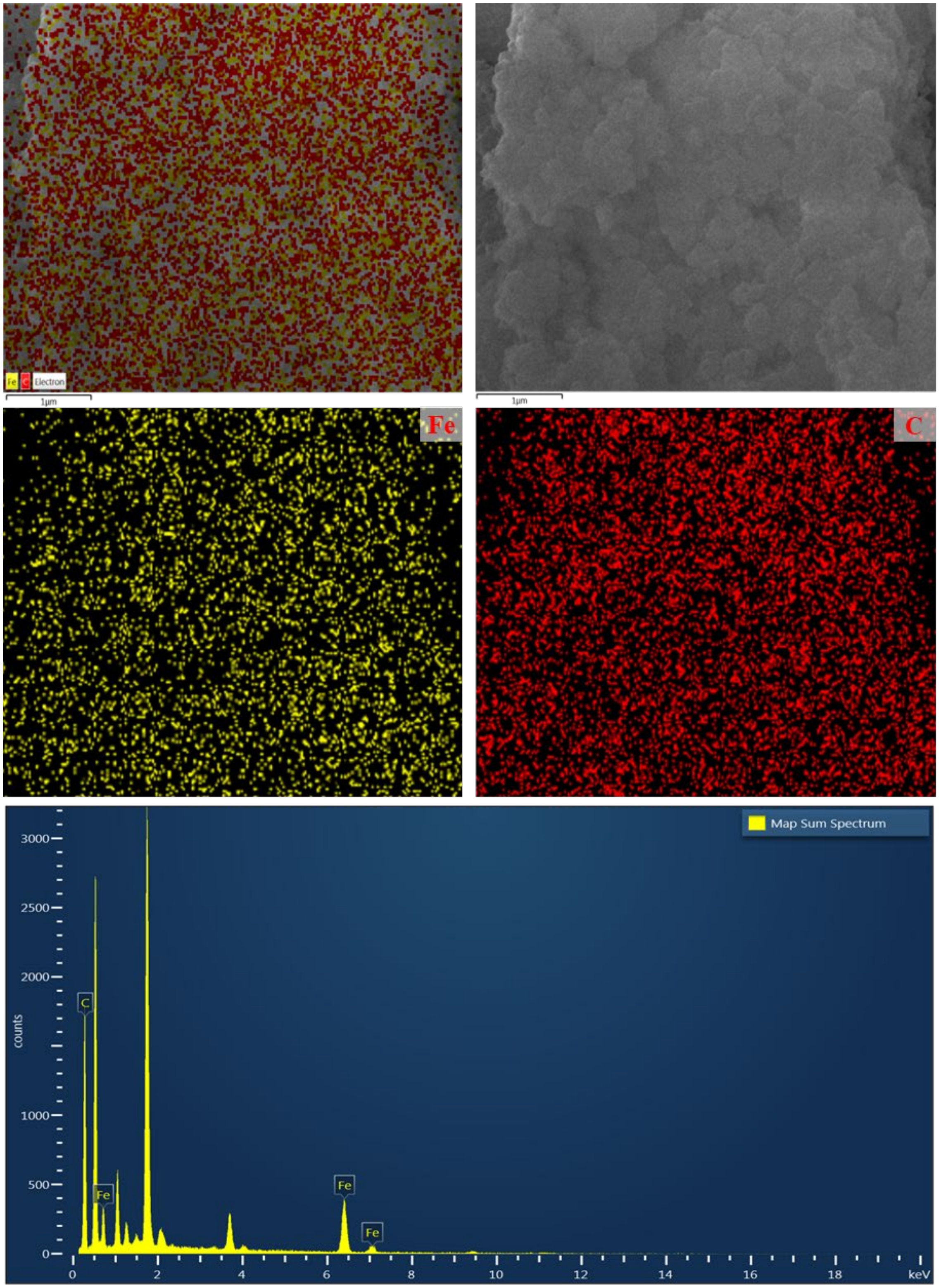


**Figure S2:** EDX mapping of elemental Fe and C on the M-100Fe@Fe2O3#1.80 sample

# References


1. Le, V. N.; Nguyen, V. C.; Nguyen, H. T.; Tran, H. D.; Tu, T. N.; Kim, W.-S.; Kim, J. *Microporous Mesoporous Mater.* **2023,** *360*, 112716.

2. Le, V. N.; Vo, T. K.; Yoo, K. S.; Kim, J. *Sep. Purif. Technol.* **2021,** *274*, 119079.

3. Le, V. N.; Vo, T. K.; Lee, J. H.; Kim, J. C.; Kim, T.-H.; Oh, K. H.; Bae, Y.-S.; Kwak, S. K.; Kim, J. *Chem. Eng. J.* **2021,** *404*, 126492.

4. Peng, J.; Xian, S.; Xiao, J.; Huang, Y.; Xia, Q.; Wang, H.; Li, Z. *Chem. Eng. J.* **2015,** *270*, 282-289.

5. Li, Y.; Yi, H.; Tang, X.; Li, F.; Yuan, Q. *Chem. Eng. J.* **2013,** *229*, 50-56.

6. Chanapattharapol, K. C.; Krachuamram, S.; Youngme, S. *Microporous Mesoporous Mater.* **2017,** *245*, 8-15.

7. Chen, C.; Ahn, W.-S. *Chem. Eng. J.* **2011,** *166*, 646-651.

8. McEwen, J.; Hayman, J.-D.; Ozgur Yazaydin, A. *Chem. Phys.* **2013,** *412*, 72-76.

9. Banerjee, R.; Furukawa, H.; Britt, D.; Knobler, C.; O'Keeffe, M.; Yaghi, O. M. *J. Am. Chem. Soc.* **2009,** *131*, 3875-3877.

10. Hu, Z.; Khurana, M.; Seah, Y. H.; Zhang, M.; Guo, Z.; Zhao, D. *Chem. Eng. Sci.* **2015,** *124*, 61-69.

11. Liang, W.; Coghlan, C. J.; Ragon, F.; Rubio-Martinez, M.; D'Alessandro, D. M.; Babarao, R. *Dalton Trans.* **2016,** *45*, 4496-4500.

12. Koutsianos, A.; Kazimierska, E.; Barron, A. R.; Taddei, M.; Andreoli, E. *Dalton Trans.* **2019,** *48*, 3349-3359.

13. Wang, Y.; Li, L.; Yan, L.; Cao, L.; Dai, P.; Gu, X.; Zhao, X. *Chin. Chem. Lett.* **2018,** *29*, 849-853.

14. Le, V. N.; Kwon, H. T.; Vo, T. K.; Kim, J.-H.; Kim, W.-S.; Kim, J. *Mater. Chem. Phys.* **2020,** *253*, 123278.

15. Mutyala, S.; Yakout, S. M.; Ibrahim, S. S.; Jonnalagadda, M.; Mitta, H. *New J. Chem.* **2019,** *43*, 9725-9731.

16. Ben-Mansour, R.; Qasem, N. A. A.; Habib, M. A. *Int. J. Energy Environ. Eng.* **2018,** *9*, 169-185.
